# Iron and ferroptosis in kidney disease: molecular and metabolic mechanisms

**DOI:** 10.3389/fimmu.2025.1531577

**Published:** 2025-02-05

**Authors:** Wenjie Wang, Jingdi Chen, Liying Zhan, Handong Zou, Lu Wang, Mengmeng Guo, Hang Gao, Jing Xu, Wei Wu

**Affiliations:** ^1^ Department of Thoracic Surgery, Renmin Hospital of Wuhan University, Wuhan, Hubei, China; ^2^ Department of orthopedics, The Airborne Military Hospital, Wuhan, Hubei, China; ^3^ Department of Critical Care Medicine, Renmin Hospital of Wuhan University, Wuhan, Hubei, China; ^4^ The First Clinical College of Wuhan University, Wuhan, Hubei, China

**Keywords:** kidney disease, ferroptosis, mechanism, treatment, metabolic

## Abstract

Maintaining iron homeostasis is necessary for kidney functioning. There is more and more research indicating that kidney disease is often caused by iron imbalance. Over the past decade, ferroptosis’ role in mediating the development and progression of renal disorders, such as acute kidney injury (renal ischemia-reperfusion injury, drug-induced acute kidney injury, severe acute pancreatitis induced acute kidney injury and sepsis-associated acute kidney injury), chronic kidney disease (diabetic nephropathy, renal fibrosis, autosomal dominant polycystic kidney disease) and renal cell carcinoma, has come into focus. Thus, knowing kidney iron metabolism and ferroptosis regulation may enhance disease therapy. In this review, we discuss the metabolic and molecular mechanisms of iron signaling and ferroptosis in kidney disease. We also explore the possible targets of ferroptosis in the therapy of renal illness, as well as their existing limitations and future strategies.

## 1 Introduction

Various forms of controlled cell death, including apoptosis, necroptosis, pyroptosis, and autophagy, have been linked to the development of kidney diseases (1). In recent years, extensive research has indicated the pivotal role of ferroptosis, a non-apoptotic form of cell death that relies on iron and results in the accumulation of lipid hydroperoxides, in the pathophysiology of the progression of kidney diseases, including Acute Kidney Injury (AKI) and Chronic Kidney Disease (CKD) (1, 2).

Iron is a crucial trace element ubiquitous in almost all living organisms. It is crucial in various biological functions, such as energy metabolism, nucleotide synthesis, and repair processes (3). Iron imbalance is frequent in patients (4). Prevalence rates as high as 24%-85% have been reported for chronic inflammatory conditions, such as CKD (5). Iron deficiency increases mortality in CKD patients (6). Conversely, iron accumulation leads to oxidative damage through Fenton reactions, which may lead to renal injury (7), indicating that iron negatively impacts CKD development and that iron accumulation plays a role in initiating ferroptosis. Therefore, it is essential to regulate proteins involved in iron metabolism to restore kidney iron metabolism and reduce the occurrence of ferroptosis.

Renal injury is distinguished by increased generation of mitochondrial reactive oxygen species (ROS) and disrupted iron homeostasis (8). Imbalances in the redox systems can result in the oxidation of specific biomolecules, thereby inducing structural and anatomical changes in these molecules. The mitochondrial cytochrome oxidase enzymes, such as cytochrome P450, regulate this process, which takes place in the mitochondria (9). Research conducted on mice models by Su et al. (10) and Zhu et al. (11) revealed that mitochondrial dysfunction precedes the onset of proteinuria and podocyte effacement. The production of ROS, metabolic byproducts that occur during this process, contributes to the development of atherosclerosis in CKD and accelerates renal damage progression.

The available evidence strongly suggests that ferroptosis has a vital function in advancing different kidney disorders. Numerous studies have identified ferroptosis as a primary cause of impaired kidney repair and the development of inflammatory responses in proximal tubular cells (12). Moreover, excessive ferroptosis, regulated by various signaling and metabolic pathways, has demonstrated its involvement in developing AKI, CKD, and renal cell cancer (RCC). This review elucidates the complex mechanisms of regulating iron homeostasis, glutathione (GSH) synthesis and lipid metabolism in the kidney. Additionally, we explore the latest indicators of ferroptosis in renal diseases and offer invaluable insights into the potential for developing novel therapeutic interventions to inhibit ferroptosis in the kidney.

## 2 Development of ferroptosis

**Figure 1:**
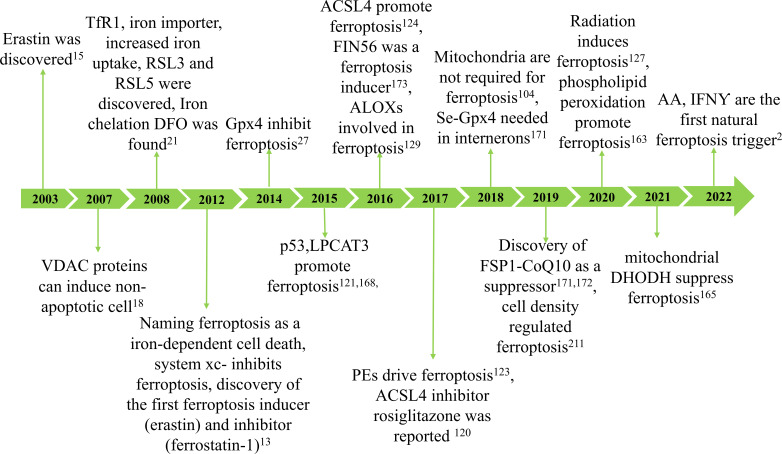
The development of ferroptosis. The concept of ferroptosis originated in 2003 with the discovery that erastin can induce a unique form of cell death characterized by the absence of traditional caspase-3 activation, DNA fragmentation, and preservation of nuclear morphology. Due to its iron dependency, this phenomenon was officially termed ferroptosis in 2012. Key hallmarks of ferroptosis include lipid peroxidation and iron accumulation, which result in diminished glutathione peroxidase (GPX4) activity and subsequent cell death. Ferroptosis arises from reduced GPX4 activity, leading to decreased cellular antioxidant capacity and the buildup of lipid reactive oxygen species (ROS). Research has demonstrated that ferroptosis is closely associated with the pathogenesis and progression of various diseases, including ischemia-reperfusion injury, kidney damage, neurological disorders, cancer, and hematological conditions.

Ferroptosis was initially reported in 2012 as a new type of cell death that can be inhibited by the iron-chelating compound deferoxamine (13). However, numerous types of cell death associated with iron and oxidative stress have been recognized for years (14). In 2003, researchers made a significant discovery related to a unique form of non-apoptotic cell death, later termed “ferroptosis” (15). Subsequent studies determined that this cell death is regulated iron-dependent (16) and is highly modulatable with molecular perturbations (17, 18). Subsequently, Marcus Conrad and his colleagues demonstrated that genetic modulation of crucial genes regulating redox state induced non-apoptotic cell death (19–21). Ferroptosis may have emerged due to understanding cysteine depletion-induced cancer cell death and glutamate-induced cytotoxicity (22, 23). Since its discovery, ferroptosis has been identified as an iron-dependent form of programmed cell death where the formation of lethal lipid peroxides is catalyzed by iron (24) (**Figure 1**).

## 3 Molecular and metabolic drivers of ferroptosis

Ferroptosis cells exhibit distinct genetic, biochemical, morphological, and metabolic characteristics compared to other recognized forms of cell death, including apoptosis, necroptosis and pyroptosis (25). In contrast to other forms of cell death, ferroptosis can rapidly propagate within cell populations in a wave-like manner (26). Regarding morphological alterations, electron microscopy can be used to observe mitochondrial abnormalities in cells undergoing ferroptosis. These changes include swelling, density variations and outer membrane rupture (27). Finally, the pathways involving iron, glutathione and lipid metabolism intersect to regulate the onset and progression of ferroptosis, particularly in renal tubular epithelial cells. The following section discusses the impact of these metabolic pathways on ferroptosis and kidney disease.

### 3.1 Iron metabolism and ferroptosis in the kidney

#### 3.1.1 modulation of iron homeostasis in the renal system

Cellular iron homeostasis is closely connected to iron absorption, storage, circulation and utilization (28). The human digestive system typically absorbs 1-2mg of iron daily from the diet (29). With the aid of cytochrome b on the brush boundary, dietary ferric iron is converted to catalytically active ferrous iron (30), which is then absorbed by intestinal epithelial cells through the assistance of the divalent metal transporter 1 (DMT1, SLC11A2) (31).

The kidney receives 20% to 25% of the overall cardiac output and is primarily a metabolic organ (32). Iron is both filtered and reabsorbed in the kidney (33). Renal cells can uptake transferrin-bound iron (TBI) and non-transferrin-bound iron (NTBI). The transferrin receptor 1 (TfR1) is expressed on both proximal and distal tubule epithelial cells, enabling the absorption of TBI (34). Transferrin is endocytosed as an additional method for TBI uptake (35). The endosome undergoes acidification, a process mediated by vacuolar ATPase, which results in the conversion of ferric iron to ferrous iron by the six transmembrane epithelial antigens of prostate 3 (STEAP3) (36, 37). Natural resistance-associated macrophage protein 2 (NRAMP2) (also called DMT1) aids in the transfer of ferrous iron from the endosome to the cytoplasm (38, 39).

The uptake of NTBI in the kidneys occurs independently of transferrin. The reabsorption of NTBI is achieved by utilizing other iron transporters, such as DMT1 (40). Zinc transporters ZIP8 and ZIP14, which were initially identified as zinc transporters, are expressed in the proximal and distal tubules and potentially contribute to the uptake of NTBI (41). These transporters can directly transport divalent iron (Fe^2+^) in the form of NTBI through the apical plasma membrane. In addition to these iron transporters, there are several additional receptors and transporters that facilitate the renal iron reabsorption process (42). Regarding the absorption of hemoglobin (Hb) and TBI, proximal tubule epithelial cells employ the megalin-cubilin complex, while distal tubule epithelial cells utilize the neutrophil gelatinase-associated lipocalin receptor (NGALR, also known as SLC22A17) (33). Both megalin and NGALR have been connected to the tubular absorption of filtered Hb during hemolysis (43).

In general, imported ferrous iron can be preserved in the cytosol within the ferritin complex, comprised of the heavy chain (Fth) and ferritin light chain (Ftl) (44). This mechanism contributes to stabilizing volatile iron concentrations and mitigates the production of ROS (45). Ferritinophagy also called the degradation of iron-saturated ferritin, is controlled by Nuclear receptor coactivator 4 (NCOA4) (46). Ferritinophagy triggers the degradation of intracellular ferritin within lysosomes, leading to the subsequent release of iron (47), This liberated iron is transported from lysosomes to the cytosol through lysosomal NRAMP2 (38). Ferritin may play a role in protecting renal cells against cisplatin-induced toxicity by regulating iron levels. Increased ferritin levels can store iron and mitigate the harmful effects of cisplatin on the kidneys. Conversely, the silence of Fth may cause cell death (48). Subsequent studies have identified that ferritin degradation is linked to the activation of NCOA4-mediated ferritinophagy. Inhibiting NCOA4-mediated ferritinophagy can effectively hinder ferritin degradation and potentially prevent kidney injury (49). Ferritin and NCOA4 play a vital role in regulating iron levels in the kidney. Moreover, a limited amount of ferrous iron is preserved in the labile iron pool (LIP), which supports cellular metabolism under normal physiological conditions (50).

Ferroportin (Fpn), the identified iron export protein in mammals, is responsible for transporting iron into the bloodstream to fulfill the needs of the organism (51–53). However, Fpn is only expressed in the epithelial cells of the proximal tubule, which plays a vital role in iron export (54). Given that Fpn is not expressed in distal tubules and these cells lack additional iron exporters, it appears that distal tubules do not have a notable function in the reabsorption and recycling of iron under normal physiological conditions (33).

**Figure 2:**
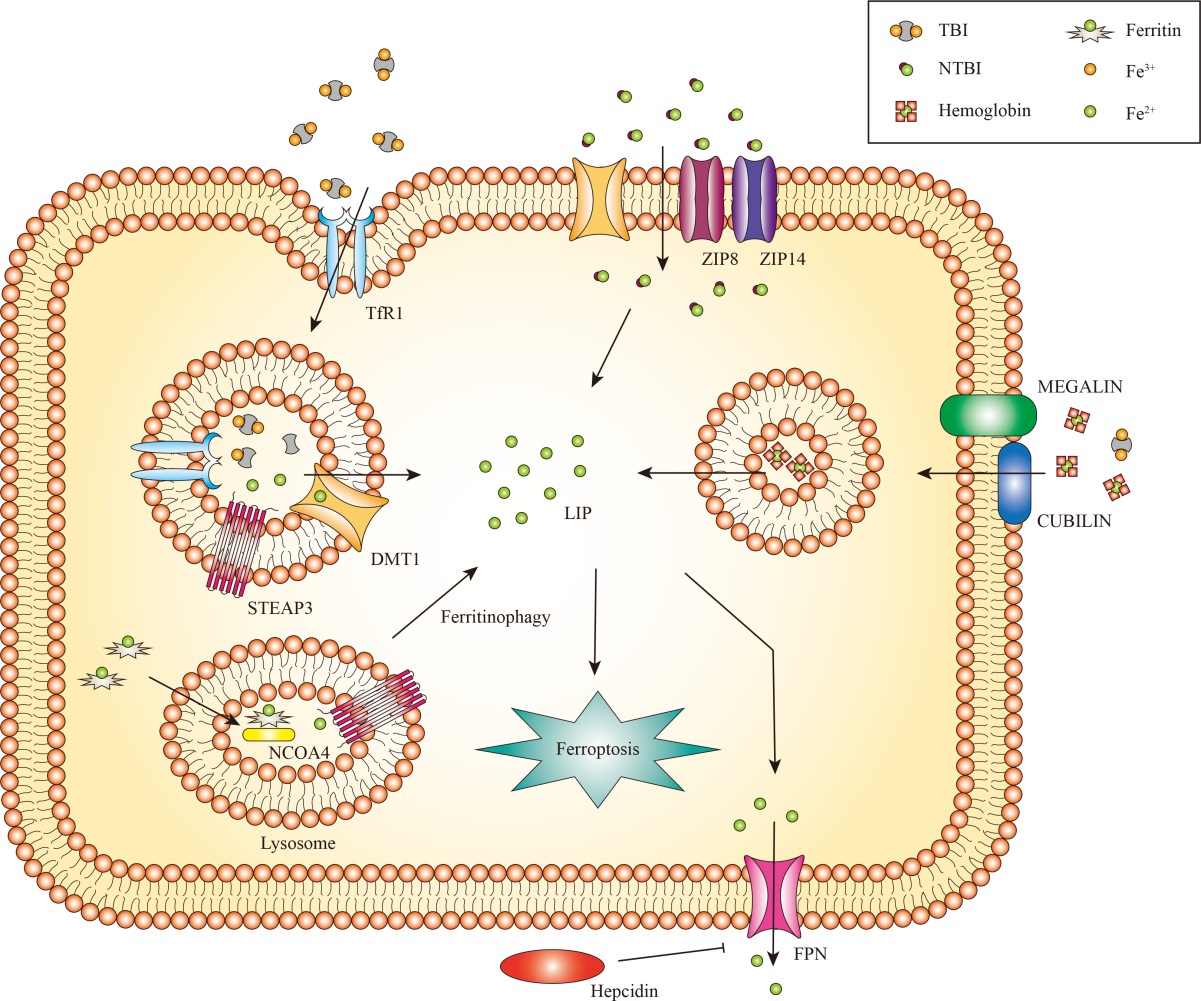
The metabolism of renal iron. In glomerular endothelial cells, endocytic uptake of TBI has been demonstrated, likely involving DMT1. Proximal and distal tubule epithelial cells reabsorb TBI from the tubular lumen through TfR1 mediated endocytosis. Divalent iron [Fe(II)] is transported into the cytosol by DMT1, zinc transporter ZIP8 and/or ZIP14. Proximal tubule epithelial cells also take up Hb and TBI through the megalin-cubilin complex. Iron can be exported to the cytoplasm by NRAMP2 after a metalloreductase STEAP3-mediated reduction. In addition, iron can be released from FTH via NCOA4-mediated autophagic degradation of ferritin, a process known as ferritinophagy. Iron export might occur via FPN. Only the proximal tubule epithelial cells express FPN for iron export, which can be inhibited by circulating hepcidin.

Fpn significantly impacts the maintenance of plasma iron levels. When the ferroportin1 (FPN1) gene, which encodes Fpn, was knocked out in AKI mice, it improved renal function by regulating iron levels (55). Hepcidin, a factor that protects against AKI, plays a central role in regulating iron homeostasis through Fpn. Hepcidin exerts its anti-kidney damage properties by promoting the ubiquitination of ferroportin through the E3 ubiquitin-protein ligase RNF21 (56), which is associated with the restoration of iron homeostasis and the suppression of inflammation (57). Hepcidin pretreatment reduces inflammation in AKI animal models and inhibits renal ischemia-reperfusion injury (IRI)-induced dysregulation of systemic iron homeostasis (58). The hepcidin-ferroportin pathway may be a promising novel therapeutic indicator for the treatment of AKI (56, 57). (**Figure 2**).

While the glomerulus can filter iron and haem proteins, there is currently limited knowledge about how glomerular cells process iron. A research study demonstrated that podocytes can uptake Hb via megalin-cubilin endocytosis, which can be metabolized by heme oxygenase 1 (HMOX1/HO-1) (59). Podocytes also contain ferritin and can take up transferrin via endocytic processes, indicating that these cells can transport and process iron (58–60). Cultured human glomerular endothelial cells have been shown to express TfR1, Fpn and DMT1 (61). Additionally, both rat and mouse mesangial cells express ferritin, TfR1 and iron regulatory protein 1 (IRP1). The induction of HO1 has been observed in these cells, indicating the involvement of iron transport and regulation (62).

The kidneys are iron-sensitive (57). During the iron cycle, NTBI and unstable ferrous irons can undergo oxidation and reduction through Fenton and Haber-Weiss reactions, resulting in oxidative damage to the kidney.

#### 3.1.2 Iron metabolism in ferroptosis

Iron is an essential trace metal element that plays a crucial role in various physiological activities, including oxygen transport, cellular respiration, DNA synthesis, and neurotransmitter biosynthesis in the nervous system (63, 64). Maintaining iron homeostasis is essential for normal cellular function, as iron deficiency and accumulation can lead to detrimental effects. Iron deficiency often leads to anemia, while an excess of iron can increase metabolic enzyme activity, such as Lipoxygenases (LOXs), which in turn enhances the generation of ROS, this process can cause tissue damage and elevate the risk of cancer (65). Therefore, iron homeostasis regulation is vital for maintaining cellular health and preventing the onset of various diseases.

The kidney has two prominent iron regulatory mechanisms: the iron-responsive element (IRE)-IRP system and the Hypoxia-inducible factor (HIF) regulatory system. The IRE-IRP1 system strictly maintains the body’s iron concentrations. The kidney expresses IRP1 and IRP2, but IRP1 is especially abundant in the proximal tubule and is vital in controlling this organ’s iron metabolism (34). In iron-depleted cells, IRP1 and IRP2 bind to IREs in the 3′ untranslated region (UTR) of target mRNAs, such as TfR1 and DMT1, leading to their stabilization and transcription of these mRNAs. On the other hand, binding of IRPs to IREs present at the 5’UTR of target mRNAs, such as Fth1 (encoding ferritin heavy chain), FPN1 and HIF-2α, prevents their translation (66, 67). This mechanism helps maintain the balance of LIPs.

HIF proteins also regulate gene transcription in cellular iron regulation (68). HIF-1α is expressed in tubular cells, whereas HIF2α is restricted to renal endothelial and interstitial cells (69). HIF-2 regulates the production of erythropoietin (EPO) in hypoxic renal interstitial fibroblast-like cells, and this process is susceptible to inhibition by IRP1 (67, 70). The regulatory role of HIF on proteins involved in iron regulation in kidney cells remains unclear. Furthermore, different proteins controlling iron balance within cells can impact the cell’s vulnerability to ferroptosis (71) (**Table 1**).

**Table 1:** Main proteins modulating iron metabolism in ferroptosis.

Gene	Protein	Location	Function	Effects of genetic deletion or overexpression	Refs.
CISD1	CDGSH iron–sulfur domain- containing protein 1 (also known as mitoNEET)	Outer mitochondrial membrane and cytoplasm	Inhibiting mitochondrial iron uptake	Deletion promotes erastin-induced ferroptosis	(72)
CISD2	CDGSH iron–sulfur domain- containing protein 2 (also known as NAF1)	ER and mitochondria associated ER membranes	Regulating mitochondrial iron homeostasis	Deletion promotes sulfasalazine- induced ferroptosis	(73)
CP	Ceruloplasmin	Cellular membranes, extracellular	Oxidizing Fe2+ to Fe3+ to mediate FPN effect	deletion promotes erastin-induced ferroptosis	(24)
SLC11A2	Natural resistance-associated macrophage protein 2	Cellular membranes, endosomal membranes	Iron importer	Deletion suppresses ferroptosis	(31)
FTH1	Ferritin heavy chain 1	Cytoplasm	Storing Iron	Deletion promotes ferroptosis in AKI	(33)
FTMT	Ferritin mitochondrial	Mitochondria	Mitochondrial iron storage	overexpression of FTMT inhibits ferroptosis	(74)
HMOX1	Heme oxygenase 1	Cytoplasm, the Golgi apparatus	Catabolizes heme	overexpression inhibits ferroptosis	(75, 76)
IREB2	Iron-responsive element binding protein 2	Cytoplasm	Regulates the translation of mRNAs that affect iron homeostasis	Deletion suppresses erastin-induced ferroptosis	(13)
NCOA4	Nuclear receptor coactivator 4	Cytoplasm	Regulates ferritinophagy	Deletion suppresses erastin-induced ferroptosis	(77, 78)
PCBP1	Poly(rC)- binding protein 1	Cytoplasm	Iron chaperones	Deletion increases ferroptosis	(79)
PROM2	Prominin 2	Multivesicular bodies	Regulates ferritin export	Deletion promotes ferroptosis	(80)
SLC25A28	Mitoferrin 2	Outer mitochondrial membrane	Regulates mitochondrial iron uptake	Deletion suppresses erastin- induced ferroptosis	(81)
SLC39A8	Solute carrier family 39 member 8 (also known as ZIP8)	Cellular membranes	Mediating the cellular uptake of Fe2+	Deletion suppresses ferroptosis	(41)
SLC39A14	Solute carrier family 39 member 14 (also known as ZIP14)	Cellular membranes	Mediating the cellular uptake of Fe2+	Deletion suppresses ferroptosis in diabetic nephropathy	(82)
SLC40A1	Solute carrier family 40 member 1(also known as ferroportin/FPN)	Cellular membranes	Mediates iron export	Deletion promotes siramesine and lapatinib-induced ferroptosis	(83)
TF	transferrin	Extracellular, endosome	Transporting iron	Deletion promotes ferroptosis	(34)
TFR1	Transferrin receptor protein 1	Extracellular, endosome	Transports iron	Deletion suppresses ferroptosis	(84)
Steap3	six transmembrane epithelial antigen of prostate 3	Cellular membranes, endosome	Mediating iron uptake	Deletion promotes ferroptosis	(36, 37)

Impairment of the uptake, transportation, storage, and utilization of iron within cells can result in an excessive accumulation of free divalent iron. This accumulation can trigger the Fenton reaction, generating hydroxyl radicals (53) and ROS (85). Subsequently, a cascade of peroxidation processes occurs on the cell membrane, involving polyunsaturated fatty acids (PUFAs), resulting in the generation of lipid peroxides, which in turn degrade the structure of the cell membrane, ultimately triggering cell ferroptosis (86).

Iron dysregulation is primarily caused by the abnormal barrier function of iron transporters Fpn (87), TfR1 and DMT1 (88). Thus, the proteins responsible for regulating the transportation of cellular iron could influence a cell’s vulnerability to ferroptosis (89).

Firstly, the deletion of TfR1 inhibits the development of renal fibrosis induced by unilateral ureteral obstruction (UUO) in a mouse model of diabetic kidney disease (DKD) by regulating iron import and suppressing ferroptosis (84). Furthermore, the induction of ferroptosis in breast cancer cells by siramesine and lapatinib is hindered by the overexpression of Fpn, which regulates iron efflux. Conversely, silencing Fpn has the opposite effect (83). Alternatively, prominin 2 (PROM2), a membrane glycoprotein, produces ferroptosis resistance in epithelial and breast cancer cells by promoting exosome-dependent iron export (80). Furthermore, CDGSH iron-sulfur domain-containing protein 1 (CISD1), also known as mitoNEET, is involved in transporting iron within mitochondria and maintaining iron and ROS balance in the outer mitochondrial membrane (90, 91). Limiting the uptake of mitochondrial iron, the upregulation of CISD1 expression protects human hepatocellular carcinoma cells against elastin-induced ferroptosis (72). CISD2 has been linked to developing resistance to sulfasalazine-induced ferroptosis in head and neck cancer. Reducing CISD2 enhances the accumulation of mitochondrial iron and restores the sensitivity of ferroptosis-resistant cells to sulfasalazine-induced cell death (73). Therefore, modulation iron transport pathway on cell membranes can regulate ferroptosis.

Alternatively, NCOA4-mediated ferritin degradation increases iron storage and accumulation in the active LIP. This triggers the Fenton reaction, resulting in mitochondrial damage, activation of LOXs function, and a rise in ROS levels (92), ultimately leading to ferroptosis. Inhibition of NCOA4-mediated ferritinophagy reduces iron storage and prevents ferroptosis in cancer cells (77, 78). Furthermore, Poly(rC)-binding protein 1 (PCBP1), an iron chaperone, interacted with NCOA4 in regulating iron flux into and out of ferritin (93). The levels of labile iron and lipid peroxidation increased in the PCBP1 knockout mice, indicating that PCBP1 may have a crucial function in preventing diseases associated with ferroptosis (79). Moreover, the overexpression of mitochondrial ferritin (FTMT), a mitochondria iron-storage protein, inhibits ferroptosis in neuroblastoma cells (74), indicating an essential antiferroptotic role in mitochondria.

Genes related to iron metabolism also can regulate ferroptosis. TfR1, Fth1 and Ftl expression during ferroptosis is influenced by the iron metabolism regulator iron-responsive element binding protein 2 (IREB2). The silencing of IREB2 affects the expression of iron-regulated genes TfR1, Fth1 and Ftl during ferroptosis (13). Furthermore, Nuclear factor erythroid 2-related factor 2 (NFE2L2) is a regulatory protein that influences heme and iron metabolism by increasing the transcription of various genes, including HO-1. HO-1 contributes to iron homeostasis (94). However, HO-1 has a dual function in ferroptosis (95, 96). The dual mechanism of protection versus cytotoxicity of HO-1 has already been confirmed by Suttner DM and Dennery PA (97). Non-canonical ferroptosis is caused by excessive HO-1 activity (98). Additionally, the serine/threonine kinase ATM enhances ferroptosis by blocking the movement of metal regulatory transcription factor 1 (MTF1) into the nucleus, which triggers the synthesis of Fpn, Fth1 and Ftl, ultimately resulting in a reduction in iron toxicity (87).

These data demonstrate that various variables influence iron metabolism during ferroptosis (4). Therefore, further investigation is required.

#### 3.1.3 Iron associated kidney disorders

Disturbances in iron homeostasis have significant impacts on the function of various organs. Iron overload, iron deficiency and imbalances in iron distribution impact iron-utilizing and recycling. As advancements in treatments for iron disorders have improved, reducing major complications and increasing patient survival, new complications are emerging, such as renal damage (99, 100).

##### 3.1.3.1 Systemic iron overload

###### 3.1.3.1.1 Hereditary haemochromatosis

Hereditary hemochromatosis (HH) is a prevalent condition characterized by excessive iron accumulation throughout the body, resulting from either hepcidin deficiency or hepcidin resistance. This condition is caused by inherited defects in genes encoding proteins involved in hepcidin production (HAMP), function (Fpn), or regulation [hereditary hemochromatosis protein (HFE), TfR2 and haemojuvelin (HJV)] (9), resulting in systemic iron overload and ferroptosis. White individuals primarily develop hereditary hemochromatosis due to a homozygous alteration in the HFE gene, which is situated on chromosome 6. Iron deposition in the kidneys and iron leakage into the urine was observed in mouse models of hemochromatosis, suggesting impaired kidney function (101). Therapeutic phlebotomy is the standard treatment for HH, involving the removal of a significant quantity of iron from erythrocytes (75, 102). This intervention leads to a progressive depletion of iron reserves in the organism.

###### 3.1.3.1.2 Halassaemia syndromes

Reduced production of α-globin or β-globin chains leads to α-thalassaemia or β-thalassaemia, respectively, which disrupts hemoglobin synthesis and subsequently impairs erythropoiesis (103). Patients with β-thalassaemia may exhibit diverse mutations and have different treatment needs (104). In both β-thalassaemia intermedia and major, ineffective and increased erythropoiesis results in reduced hepatic hepcidin production (105). Frequent red blood cell (RBC) transfusions for the treatment of anemia and the inherited defect exacerbate systemic iron overload (104). Iron removal therapy in patients with β-thalassaemia involves the treatment of iron chelation medication. β-thalassaemia has been associated with increased renal iron exposure, as reflected by increased urinary iron excretion, deposition, and kidney injury. Renal injury and iron accumulation are frequently observed in patients with β-thalassaemia. These patients may exhibit elevated levels of urinary markers for kidney injury, including cystatin C, β2-microglobulin and N-acetyl-β-d glucosaminidase, in the absence of kidney failure (106–109).

##### 3.1.3.2 Systemic iron deficiency

Iron deficiency disorders can be classified into two types: absolute and functional iron deficiency (FID), both of which can result in anemia. Iron deficiency anemia (IDA) is most common in women of childbearing age, pregnant women and children (5). In these individuals, insufficient body iron stores result from inadequate dietary iron intake, increased iron need, or iron loss, which hampers the support for erythropoietic needs. Anemia due to FID can be inherited or acquired. Mutations in TMPRSS6, which encodes matriptase-2, have been found to result in the iron-refractory IDA (IRIDA) (110). The mechanisms of matriptase-2 mediated inhibition of hepcidin synthesis have yet to be resolved entirely. Matriptase-2 cleaves various components in hepcidin-activating pathways, including HJV, Activin receptor-like kinase 2 (ALK2) and HFE. However, in patients with IRIDA, matriptase-2 is non-functional, leading to elevated hepcidin levels. These high levels of hepcidin impede the absorption of dietary iron and the release of iron from macrophages (111, 112). Acquired FID frequently occurs in patients with elevated levels of inflammation, including individuals with systemic inflammatory disorders such as CKD or cancer. This condition is also known as anemia of inflammation or anemia of chronic disease (110). Iron levels can be restored through oral or IV iron supplementation for patients with IDA or FID. However, individuals with FID may require additional therapy to stimulate erythropoiesis and decrease hepcidin levels (113, 114).

### 3.2 GSH metabolism and ferroptosis in the kidney

Glutamate and glutamine are also important regulators of ferroptosis (115). System xc- (also known as xCT) facilitates the exchange of glutamate and cystine in equal proportions. It is a cystine-glutamate antiporter system, with SLC7A11 as its active subunit (116). The function of system xc -is affected by glutamate levels. Indeed, elevated levels of extracellular glutamate concentrations inhibit system xc- and induce ferroptosis (13). Knockout mice lacking system xc- knockout mice exhibit protection against brain damage due to the reduction of extracellular brain glutamate levels (117). Thus, the concentration of extracellular glutamate may induce ferroptosis.

System xc^-^ regulated cystine uptake is critical in enhancing GSH biosynthesis (118). The entry of cystine into the cell affects the production of cysteine and GSH (13). GSH activates glutathione peroxidase 4 (GPX4), significantly influencing intracellular redox homeostasis maintenance. By inhibiting system xc- activity, sulfasalazine and sorafenib can disrupt cystine absorption and GSH synthesis, leading to a reduction in GPX4 activity and cell antioxidant capacities, an increase in lipid ROS accumulation, and the gradual onset of oxidative damage and ferroptosis. By utilizing mice with inducible GPX4 knockout, we have uncovered a crucial function of the GSH/Gpx4 axis in preventing sudden kidney malfunction and the related occurrence of ferroptosis (27).

Human tissues and plasma contain glutamine in its natural state. The glutaminolysis of glutamine can promote the tricarboxylic acid (TCA) cycle and lipid biosynthesis processes. The absence of glutamine or glutaminolysis inhibition hampers the accumulation of ROS, thereby preventing lipid peroxidation and ferroptosis. This finding can be explained by the requirement of α-ketoglutarate (αKG), a product of glutaminolysis, for ferroptosis (115).

Since ferroptosis has been recognized as a significant mechanism of cell death in tissue injury (119, 120), targeting glutaminolysis may emerge as a practical therapeutic approach for managing organ damage mediated by ferroptosis. Indeed, studies have demonstrated that inhibiting glutaminolysis can reduce kidney damage induced by ischemia/reperfusion (I/R) in experimental models (120).

### 3.3 Lipid metabolism and ferroptosis in the kidney

Ferroptosis is characterized by uncontrolled oxidation of lipids (121). The oxidation of PUFAs in the membranes of mammalian cells is a crucial process in ferroptosis (122). Genome-wide and CRISPR/Cas9-based screens have revealed that Acyl-CoA synthetase long-chain family member 4 (ACSL4) and lysophosphatidylcholine acyltransferase 3 (LPCAT3) (123–125), both membrane-remodeling enzymes, are significant contributors to the induction of ferroptosis. Upregulation of ACSL4 increases the content of PUFAs in phospholipids, thereby rendering the cell more susceptible to ferroptosis (123, 126). Although the role of ACSL4 in the kidney is unclear, increased expression and activity of ACSL4 might promote ferroptosis under various pathophysiological conditions (127). Identifying this enzyme as a potential therapeutic target shows promise in reducing cell death in skeletal muscle and preventing rhabdomyolysis (128). Another study demonstrated a close association between ferroptosis and intestinal IRI, highlighting the critical role of ACSL4 in this lethal process (129). Furthermore, ionizing radiation can trigger the activation of ACSL4 and facilitate ferroptosis in malignant cells. Deleting ACSL4 significantly eliminates ionizing radiation-induced ferroptosis and promotes radioresistance, thus highlighting the crucial role of ACSL4 in cancer treatment (130). By contrast, ACSL3 catalyzes the conversion of exogenous monounsaturated fatty acids (MUFAs) to fatty acyl-CoAs and inhibits ferroptosis (131, 132).

LOXs, a group of iron-containing enzymes, can directly oxygenate PUFAs in biological membranes (133), indicating that LOXs may mediate lipid peroxidation in ferroptosis. This hypothesis is supported by the evidence that certain pharmacological inhibitors of LOX can inhibit ferroptosis (134, 135). 12/15-LOX knockout protected mice against ischemic brain injury (136, 137). However, the genetic removal of 12/15-LOX on the GPX4 knockout background failed to prevent ferroptosis in mouse AKI (27). The results indicate that alternative mechanisms compensate for LOX loss (138). Furthermore, it has been demonstrated that the scaffold protein PEBP1 can bind and guide 15-LOX toward PUFAs in the membrane, thus facilitating the process of ferroptosis (139).

The kidney is extremely vulnerable to oxidative harm, and lipid peroxidation is vital in kidney injury caused by ROS. Malondialdehyde (MDA), 4-hydroxyhexenal (4-HHE) and 4-hydroxynonenal (4-HNE) are significant end products of PUFAs oxidation and are frequently utilized as indicators of lipid peroxidation (140). The average serum MDA levels were significantly increased in chronic renal failure (CRF) patients. Additionally, a high plasma level of MDA was identified as a robust and independent predictor of mortality in CRF patients (141, 142). These findings support the hypothesis that lipid peroxidation plays a role in the severity of kidney disease. Lipid peroxidation drives ferroptosis by damaging cellular membranes (143). *In vivo*, removing lipid peroxides could increase the lifespan of mice with deficiencies by around 35% (27). A certain compound has been demonstrated to inhibit the characteristic morphological alterations of ferroptosis cells by decreasing MDA and lipid ROS levels in proximal tubular epithelial cells (144). Quercetin treatment abolished lipid ROS, protecting against functional acute renal failure (144). Overall, both direct and indirect evidence from various studies strongly supports the crucial involvement of lipid peroxidation in ferroptosis.

### 3.4 Mitochondria and kidney ferroptosis

The density of mitochondria in kidneys is among the highest. Mitochondria coordinate essential metabolic processes. Dysfunction in the mitochondria may result in an energy crisis, impact the redox status of cells, and control ferroptosis. Mitochondrial defects in renal tubules contribute to the development of kidney disease by causing epithelial atrophy, inflammation and cell death (9).

Iron exerts its functions mainly by forming iron-sulphur (Fe-S) clusters and haem. Fe-S clusters are assembled from ferric iron in the mitochondria and incorporated into Fe-S proteins primarily in the mitochondria and cytosol (145, 146). Mitochondria acquire iron via mitoferrin 1 and mitoferrin 2 (81). Subsequently, iron binds to mitochondrial ferritin (gene named FTMT) to inhibit the production of ROS. Moreover, mutations in FTMT are associated with mitochondrial iron overload and cytoplasmic iron deficiency (147). Currently, the mechanism responsible for the export of iron from the mitochondria remains unknown (146). Moreover, the export of Fe-S clusters into the cytoplasm may depend on the presence of iron-sulfur clusters transporter ABCB7 and mitochondrial ABCB7 and ABCB8 (148). Additionally, it has been shown that mitochondrial ABCB10 plays a role in regulating the initial stages of haem synthesis within mitochondria (149).

Haem, acting as a cofactor in facilitating catalysis and electron transfer (150), is synthesized in the mitochondria through a complex biogenic pathway involving aminolevulinic acid synthase (ALAS, also known as ALAS-H), which plays a crucial role in the rate-limiting initial step (151). Excess haem can be transported to the cytoplasm through feline leukemia virus subgroup C receptor-related protein 1B (FLVCR1B) or catabolized via the HO-1 pathway into Fe^2+^ (152). The expression of HO-1 is ubiquitously induced in response to oxidative stress (153). In mammals, haem efflux is primarily mediated by the plasma membrane exporter FLVCR1A and membrane importer FLVCR2 (154, 155). There are other haem transporters, such as haem carrier protein 1 (HCP1) and haem responsive gene protein 1 (HRG1), but their functions need to be better understood (156).

Mitochondria are the primary source of ROS in most cells (157). Since 2012, it has been recognized that mitochondria play a crucial regulatory role in ferroptosis. Scientists observe changes in mitochondrial morphology during ferroptosis, such as decreased mitochondrial volume and elevated mitochondrial membrane density (13). Certain compounds have been specifically engineered to target mitochondria and exhibit protective effects against ferroptosis (158). In contrast, the evidence contradicts the relationship between mitochondria and ferroptosis. Previous research has indicated that a cell line with mitochondrial defects is equally susceptible to ferroptosis compared to its parental cell line. Moreover, this sensitivity can be mitigated by administering iron chelators (13, 159). However, there is significant controversy surrounding these findings. The involvement of mitochondria in ferroptosis could vary depending on the context (27, 160).

Kidney tubule cell death is the predominant phenotype observed in global GPX4 knockout mice, highlighting the crucial involvement of GPX4 and ferroptosis in kidney disease (27). Conversely, conditional GPX4 deletion promotes multiple organ dysfunction (161). The GPX4 gene in mammalian cells encodes cytosolic and mitochondrial isoforms. The mitochondrial isoform of GPX4 localizes in mitochondria due to a mitochondrial targeting signal at its amino terminus (162, 163). The cytosolic GPX4 isoform can accumulate in the mitochondrial intermembrane, playing a role in suppressing mitochondrial lipid peroxidation (162). Interestingly, the overexpression of cytosolic GPX4, rather than the mitochondrial isoform, prevented the death of GPX4 knockout mouse embryonic fibroblasts (164). Overall, these findings reveal that the expression of cytosolic GPX4 is adequate to prevent ferroptosis.

*In vivo*, experiments have also shown that mitochondria play a significant role in ferroptosis. The mitochondria ROS scavenger (MitoTEMPO) has been demonstrated to protect against ferroptosis-induced nephropathy (165). Furthermore, mice that lack FTMT encounter heightened brain injury and neurological impairments, along with characteristic molecular characteristics of ferroptosis. Conversely, FTMT overexpression reverses these changes. This research proves that FTMT is critical in protecting against cerebral I/R-induced ferroptosis and subsequent brain damage (166).

**Figure 3:**
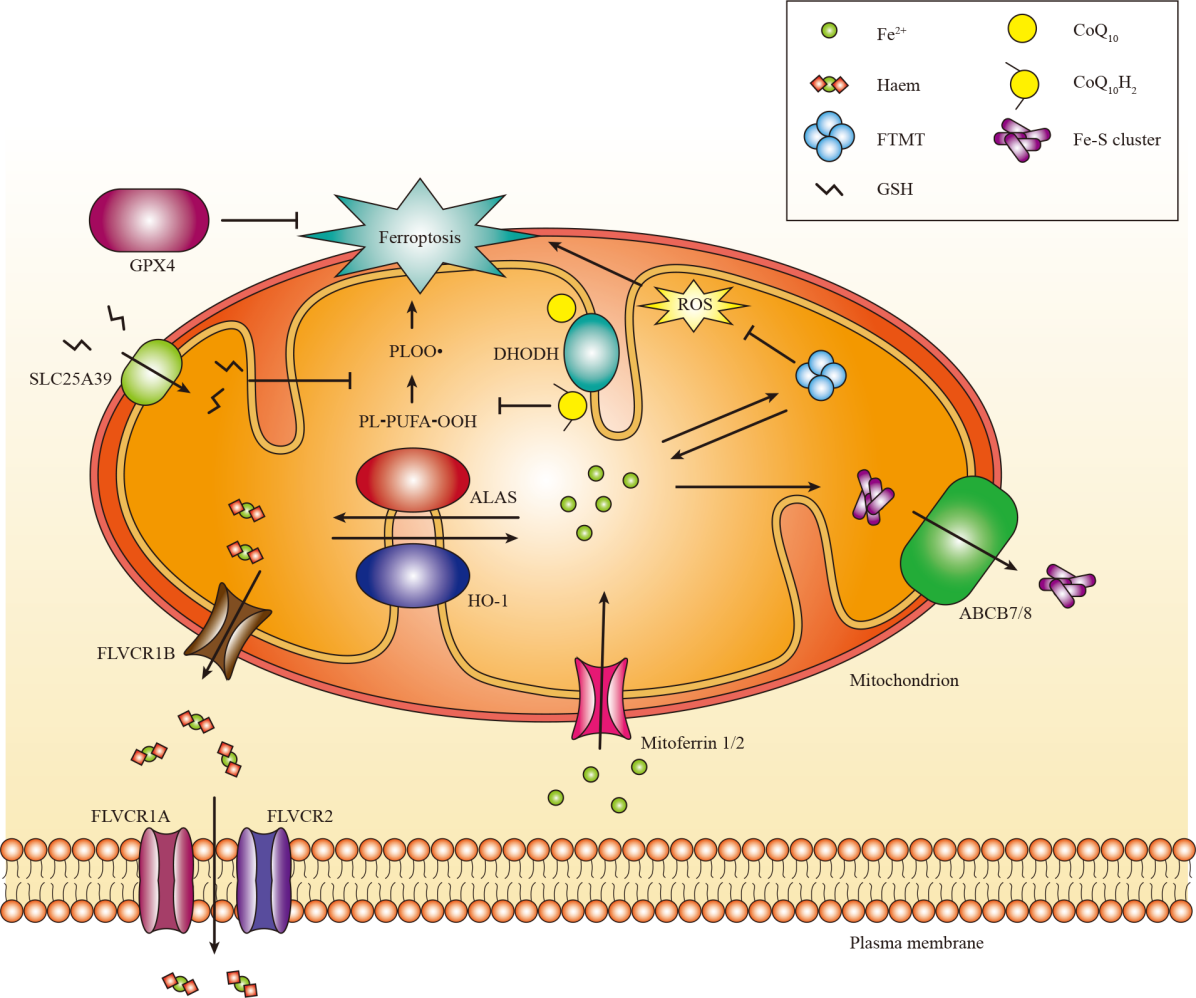
The role of mitochondria in ferroptosis. Mitochondria host a wide range of key metabolic processes of ferroptosis, such as lipid peroxidation. Separate mitochondria-localized defense systems have evolved to prevent mitochondrial lipid peroxidation and ferroptosis. For example, either the mitochondrial version of phospholipid hydroperoxide GPX4 or mitochondrial (DHODH) can specifically detoxify mitochondrial lipid peroxides. Moreover, FTMT protects mitochondria from iron overload induced oxidative injury. Iron is primarily used to Fe-S clusters and haem in the mitochondria. Excess iron can be stored in the FTMT. Mitoferrin 1 and mitoferrin 2 mediate the transport of iron across the mitochondrial membrane. FLVCR1B promotes haem efflux into the cytoplasm, whereas the export of Fe-S clusters into the cytoplasm might require Fe-S clusters transporter ABCB7 and ABCB8. PL-PUFA-OOH, polyunsaturated fatty acid-containing phospholipid hydroperoxides; PLOO·, phospholipid peroxyl radical.

Multiple studies have demonstrated that mitochondria can act as initiators or amplifiers of cystine starvation and GSH depletion induced by ferroptosis. The ability of mitochondrial dihydroorotate dehydrogenase (DHODH) and mitochondrial coenzyme Q10 (CoQ10) to suppress ferroptosis triggered in this manner (167), highlights this function of mitochondria. The localization of DHODH occurs within the inner membrane of mitochondria, facilitating dihydroorotate transformation into orotate (168). Interestingly, it has been demonstrated that DHODH inactivation coupled with low expression of GPX4 leads to a significant increase in mitochondrial lipid peroxidation and ferroptosis (167). Furthermore, the transportation of GPX4 to the mitochondria restores RSL3-induced ferroptosis in cells lacking DHODH (167). The mitochondrial GSH importer, SLC25A39, has been discovered, indicating its potential role in controlling mitochondrial lipid peroxidation and ferroptotic cell death (169). However, further investigations are necessary to elucidate the *in vivo* role of DHODH and discover other mitochondrial enzymes that regulate ferroptosis (**Figure 3**).

### 3.5 Other pathways that regulate ferroptosis

Other pathways can also regulate Ferroptosis. First, the p53 pathway is essential, which performs dual regulatory functions in ferroptosis. On the one hand, p53 can enhance ferroptosis by inhibiting the expression of SLC7A11 (170). In contrast, p53 inhibits ferroptosis by diminishing the activity of dipeptidyl peptidase-4 (DPP4) and enhancing the expression of cyclin-dependent kinase inhibitor 1A (CDKN1A) (171). Research has shown that p53 can promote ferroptosis in glomerular podocyte injury induced by high fructose (172). However, the exact role of p53 in various kidney diseases still needs to be better understood. Second, ferroptosis suppressor protein 1 (FSP1) (173, 174). The FSP1-coenzyme Q10-nicotinamide adenine dinucleotide phosphate (FSP1-CoQ10-NADPH) axis functions as a parallel system that collaborates with GPX4 and GSH to inhibit phospholipid peroxidation and suppress ferroptosis in cancer (174, 175). The absence of FSP1 makes kidneys more susceptible to tubular ferroptosis in a manner that relies on GSH, leading to a distinct morphological pattern of tubular necrosis (176). The precise role of FSP1 in the kidney still needs to be better comprehended.

## 4 Ferroptosis in kidney disease

### 4.1 AKI

AKI is a clinical syndrome marked by a sudden decrease in renal excretory function, such as the accumulation of creatinine (Cr), urea nitrogen (BUN) and other metabolic waste products. The pathogenesis of AKI is complex, involving multiple factors, including renal IRI, drug-induced AKI, severe acute pancreatitis-induced AKI, and sepsis-associated AKI. These factors are closely associated with the occurrence of ferroptosis.

#### 4.1.1 Renal IRI

Renal IRI is a severe condition contributing to AKI and substantially impacting mortality rates. Ferroptosis is responsible for the death of tubular cells, a crucial element in I/R renal injury (177). In the mouse IRI model, ferroptosis inhibitors demonstrated protective effects against AKI and other organ damage (176). Renal IRI is the primary factor responsible for AKI following cardiac surgical procedures in clinical settings. The development of postoperative AKI in the heart is intricate, with the liberation of unbound iron playing a crucial part (178). Elevated free iron levels can trigger cellular ferroptosis, resulting in tubular necrosis (179). This finding highlights the significance of maintaining iron homeostasis in AKI induced by I/R (179). In addition, I/R-induced AKI is linked to the GSH-glutathione peroxidase (GSH-GPx) system. A rat I/R kidney injury model revealed that miR-182-5p and miR-378a-3p could bind GPX4 and SLC7A11 mRNAs to inhibit their expression, which in turn activated ferroptosis to reduce I/R kidney injury (180). Another research verified that the inhibition of Legumain, which is involved in the lysosomal degradation of GPX4 during ferroptosis, mitigated both ferroptosis and tubular damage in a model of ischemia/reperfusion kidney injury by elevating intracellular GPX4 levels (181).

#### 4.1.2 Drug-induced AKI

Drugs frequently contribute to the occurrence of AKI. Folic acid (FA) can induce AKI in mice. Diego et al. found that FA-induced AKI in mice was associated with lipid peroxidation and suppression of GSH metabolism, consistent with the typical profile of ferroptosis. Moreover, administering ferrostatin-1, a ferroptosis inhibitor, decreased kidney damage and oxidative stress levels in mice with FAI. These findings suggest that ferroptosis is closely linked to the process of FA-induced AKI (182). In addition, cisplatin is a commonly used antitumor drug; however, it is nephrotoxic and can cause AKI in patients. Cisplatin induces acute tubular necrosis through DNA damage and disruption of mitochondrial function. Research indicated that iron was detectable in the medium after exposure to cisplatin. The increase in iron levels can catalyze free radical reactions while using iron chelators significantly reduces cisplatin-induced cytotoxicity. These findings suggest a crucial involvement of iron, although the precise mechanism remains elusive (183). Ferritin plays an essential role in iron metabolism. Fth1 knockout mice are more susceptible to developing AKI after cisplatin treatment, which emphasizes the protective role of Fth1 in AKI (33). Myo-inositol oxygenase (MIOX) is an enzyme to promote ferroptosis, which can inhibit GPX4 activity and increase ferritin phagocytosis. The renal pathological damage and ferroptosis levels were elevated in cisplatin-treated mice overexpressing MIOX. These findings suggest a strong association between the cisplatin-induced AKI process and ferroptosis (184).

#### 4.1.3 Severe acute pancreatitis-induced AKI

AKI is a common complication of severe acute pancreatitis (SAP) (185). The exact pathophysiology of AKI in SAP remains unclear. Previous studies have indicated that AKI induced by SAP is primarily caused by the loss and dysfunction of renal tubular epithelial cell barriers induced by oxidative stress (186). Deliang et al. conducted a rat model of SAP-induced AKI and observed an elevation in the expression of ACSL4, a positive ferroptosis regulator. However, negative regulators expressions, such as GPX4 and Fth1, were suppressed. Moreover, morphological features of ferroptosis were observed, suggesting an association between ferroptosis and SAP-induced AKI. In addition, using Liproxstatin-1, a ferroptosis inhibitor, effectively reduced histopathological damage in the pancreas and kidneys of SAP rats (187). Other studies have shown that AKI may result from various forms of cell death in SAP (188). However, further investigation is required to better understand the interconnections among these cell death processes.

#### 4.1.4 Sepsis-associated AKI (SA-AKI)

Sepsis is a major cause of AKI in intensive care units. Patients with SA-AKI experience prolonged hospital stays and have a greater mortality risk than others (189). Previous studies have demonstrated that renal tubular cells undergo necrosis, apoptosis, and autophagy in the SA-AKI model (190). Ji et al. initially confirmed the presence of ferroptosis in SA-AKI, and further experiments indicated that Nrf2, an essential protein regulating iron metabolism, was involved in inhibiting ferroptosis in the SA-AKI model (191). Similarly, Qiu et al. used Ferrostatin-1 to inhibit ferroptosis and thereby reduce SA-AKI and found that melatonin also inhibited this process. Subsequent studies have confirmed that melatonin inhibits ferroptosis by activating the Nrf2/HO-1 pathway, which is involved in the inflammatory response and antioxidant processes in SA-AKI, reducing ROS accumulation (192).

### 4.2 CKD

Due to its high incidence and mortality, CKD is a significant public health problem. The intricacy of molecular mechanisms in CKD has sparked a growing interest in this particular disease area. The pathology of CKD is significantly influenced by ferroptosis (193).

#### 4.2.1 Diabetic nephropathy (DN)

DN, one of the significant complications of diabetes, is a severe microangiopathy. The main features are the progressive decline in renal function and the increase in proteinuria. Recent findings indicate that ferroptosis plays a role in developing DN (194). The decreased expression of GPX4 and elevated lipid peroxidation were confirmed in the renal tissue of DN mice, and the administration of ferrostatin-1 reversed these changes (195). In addition, researchers have observed inhibition of Nrf2 expression in DN models. Reducing Nrf2 levels enhances the susceptibility of DN cells to ferroptosis and fenofibrate by upregulating Nrf2 expression and inhibiting ferroptosis, which can decelerate DN progression. This finding suggests a promising avenue for developing future therapeutic interventions (196). It has been verified that HMGB1 relocates to the cell’s nucleus and diminishes the Nrf2 expression along with its subsequent objectives. The potential for innovative therapeutic approaches in DN lies in targeting HMGB1 and ferroptosis (197). Furthermore, DN mice exhibited elevated lipid peroxidation levels and iron accumulation, resulting in the upregulation of ferroptosis indicator ACSL4 and the downregulation of GPX4 in renal tubular cells (76). In particular, the upregulation of HO-1 expression has been demonstrated to inhibit oxidative stress and improve redox balance, potentially offering benefits for DN (198).

#### 4.2.2 Renal fibrosis

Renal fibrosis is crucial in advancing CKD to end-stage renal disease. Its main pathological features include glomerulosclerosis, tubulointerstitial fibrosis, and excessive accumulation of extracellular matrix (1). Previous studies have demonstrated an increased expression of prostaglandin-endoperoxide synthase (PTGS2), a potential marker of ferroptosis, in animal models of renal fibrosis (199). Other ferroptosis-related molecules, such as GPX4, iron and lipid peroxides, have also been shown to be involved in ferroptosis in the pathogenesis of renal fibrosis (200). TTGF-β1 is widely recognized as a pivotal factor in developing renal fibrosis. TGF-β1 inhibits the expression of SLC7A11 and GPX4, demonstrating synergistic effects with ferrostatin-1 in inhibiting ferroptosis (195). Other studies have demonstrated that Deferasirox (DFX) may reduce renal fibrosis by inhibiting TGF-β1/Smad3, inflammation and oxidative stress pathways (201). It has also been suggested that roxadustat may impede renal fibrosis by inhibiting ferroptosis; however, further investigation is required to ascertain the specific mechanism (202).

#### 4.2.3 Autosomal dominant polycystic kidney disease (ADPKD)

ADPKD is the prevailing inherited renal disorder resulting from mutations in PKD1 (encoding polycystin-1) or PKD2 (encoding polycystin-2), which mainly presents with cysts of varying sizes in the kidneys bilaterally. The gradual enlargement of the renal cysts ultimately destroys the kidneys’ structure and function, leading to end-stage renal failure (203). The researchers observed an increase in intracellular ferroptosis in the PKD1 mutant mouse model, characterized by elevated expression of HO-1 and iron importers (TfR1, DMT1), enhanced lipid peroxidation levels, and decreased expression of GSH and GPX4. Ferroptosis inducer erastin accelerates ADPKD progression, and ferroptosis inhibitors, such as ferrostatin-1, can reduce ADPKD progression (204). Schreiber et al. used ferroptosis inducer RSL to promote ADPKD cysts growth by activating TMEN16A. Conversely, ferrostatin-1 inhibited TMEN16A expression, preventing cysts progression (205). The results reveal that ferroptosis plays a vital role in regulating ADPKD progression.

### 4.3 RCC

RCC is a malignant neoplastic disease that originates from the renal tubular epithelium. Oxidative phosphorylation and metabolic reprogramming are important features of RCC (206). Numerous studies have substantiated the essential significance of ferroptosis in RCC (207). Evidence indicates that the inhibition of ferroptosis is a common occurrence in cancer cells, enabling their survival and progression. The resistance to RCC therapy remains a significant challenge. However, it has been observed that cancer cells, resistant to conventional chemotherapeutic drugs, exhibit high susceptibility to ferroptosis inducers. Inducing ferroptosis can effectively overcome the resistance of cancer to standard therapies (208). For example, RCC can be susceptible to erastin-induced ferroptosis in mouse tumor xenograft models (209). In addition, RCC is strongly linked to mutations in the von Hippel Lindau (VHL) gene. Previous studies have found that exogenous expression of pVHL can enhance oxidative metabolism in RCC cells, thereby leading to the inhibition of ferroptosis (210). Additionally, Xu et al. found that Acyl-CoA thioesterase 8 (ACOT8) expression was significantly reduced in RCC tissues, and its high expression suggested a poor prognosis. Functional analysis revealed that ACOT8 correlated with Oxidative phosphorylation and fatty acid metabolism. Moreover, the analysis revealed a positive association between ACOT8 and GPX4 while demonstrating a negative correlation with factors that inhibit ferroptosis. These findings imply that ACOT8 potentially regulates RCC development by suppressing ferropsosis (211). Moreover, Yang et al. found that cell density affects the sensitivity of RCC cells to ferroptosis, and the Hippo-Yes-associated protein (YAP)/Transcriptional coactivator with PDZ-binding motif (TAZ) pathway is involved in this process. Meanwhile, suppression of TAZ expression inhibited ferroptosis and overexpression of TAZS89A promoted ferroptosis. TAZ regulates NADPH Oxidase 4 (NOX4) levels by regulating epithelial membrane protein 1 (EMP1) expression, essential for ROS processes in ferroptosis. The findings reveal that cell density-regulated ferroptosis is mediated by TAZ through the regulation of EMP1-NOX4, suggesting its therapeutic potential for RCC (212). Emerging evidence suggests that ncRNAs act as pivotal regulators of various cellular processes in RCC. RNA-based therapeutics offer an alternative approach to modulating ferroptosis in cancer therapy, fostering ferroptosis in cancer treatment. These findings emphasize the need for further investigation into the therapeutic potential of ncRNAs in RCC (213).

### 4.4 Other kidney-related diseases

SARS-CoV-2 virus leads to COVID-19, resulting in a range of symptoms from mild to severe in humans (214). While SARS-CoV-2 primarily induces lung damage, it is also capable of causing extrapulmonary manifestations, such as renal involvement (215). Several studies have confirmed that AKI induced by SARS-CoV-2 is associated with up to a five-fold increase in in-hospital mortality compared to patients without AKI (216, 217). Ninety percent of hospitalized patients have abnormal serum iron levels, which correlate with the severity of the disease (218). Iron metabolism dysfunction has been widely documented in many COVID-19 patients. Consequently, this dysregulation could induce ferroptosis in renal tubule cells (219, 220). Ferroptosis has been indicated as a potential therapeutic target for the treatment of COVID-19 (221). Renal dysfunction and AKI frequently manifest in patients with initially healthy kidneys during severe SARS-CoV-2 infection (222). Moreover, through the use of RNA sequencing (RNA-seq), it has been observed that cilastatin, an inhibitor of dipeptidase-1 (DPEP1), is capable of preventing SARS-CoV-2 infection and the subsequent ferroptosis (219).

Cardiorenal syndrome (CRS) encompasses a spectrum of disorders involving the heart and kidneys, where acute or chronic dysfunction in one organ can precipitate acute or chronic dysfunction in the other. This syndrome can be classified into five subtypes: Type 1 (acute CRS), Type 2 (chronic CRS), Type 3 (acute renocardiac syndrome), Type 4 (chronic renocardiac syndrome), and Type 5 (secondary CRS) (223). Empagliflozin (EMPA) is a selective inhibitor of sodium-glucose cotransporter 2 (SGLT2). It has been shown to significantly enhance cardiac and renal function by mitigating doxorubicin-induced ferroptosis, fibrosis, apoptosis, and inflammation in mice through mechanisms involving NLRP3 and MyD88-related pathways (82).

## 5 Ferroptosis as a promising treatment target

Increasing research on ferroptosis has provided mounting evidence suggesting its ability to inhibit tumor growth and enhance the efficacy of chemotherapeutic drugs (116). However, ferroptosis exerts distinct effects on different types of renal diseases. This therapeutic target shows great promise for treating and preventing kidney disease. We have compiled a summary of ferroptosis targeting agents with therapeutic potential in kidney diseases in **Table 2**.

**Table 2:** Summary of ferroptosis targeting agents with therapeutic potential in kidney diseases.

Drugs	NCT	Mechanism	Targets	Refs
Ferrostatin 1		Inhibits lipid peroxidation	I/R-induced AKI,folic acid-induced AKI	(27, 120, 181)
			lipid-oxidation-induced acute renal failure	(222)
			primary kidney renal tubule damage model	(224)
			ADPKD mice, cisplatin induced renal injury	(203, 223)
			DN	(82, 194)
UAMC-3203		Inhibits lipid peroxidation	multiorgan injury, including kidney	(225)
			renal I-R injury	(226)
liproxstatin 1		Inhibits lipid peroxidation	lipid-oxidation-induced acute renal failure	(21)
			Fe(II)-dependent renal tubular injury model	(227)
			SAP-induced AKI	(186)
Tectorigenin		Inhibits lipid peroxidation	UUO mouse model	(228)
Nec-1f		Inhibits necroptosis and ferroptosis	renal I-R injury	(175)
Deferoxamine	00870883	Inhibits iron overload	CIN	(229)
	04633889		renal tubulointerstitial fibrosis	(230)
			renal injury	(231)
Deferasirox		Inhibits iron overload	CKD rat model	(200)
			iron-induced renal injury	(232)
Deferiprone	01146925	Inhibits iron overload	CIN	(233)
	01770652		aluminum chloride-induced AKI	(234)
N-acetylcysteine	01218178		CIN	(235)
	00780962		AKI	(236–238)
SRS 16-86		Ferroptosis inhibition	renal I-R injury	(120)
rosiglitazone		ACSL4 inhibitor	I/R-induced AKI	(128)
			DN	(239)
ciclopirox olamine (CPX-O)		Inhibits iron overload	ADPKD	(240)
L-buthionine (S,R)-sulfoximine		Inhibits lipid peroxidation	RCC	(241)

I–R, ischaemia–reperfusion; DN, diabetic nephropathy; ADPKD, autosomal dominant polycystic kidney disease; SAP, severe acute pancreatitis; UUO, unilateral ureter obstruction; CIN, Cisplatin-induced nephrotoxicity.

### 5.1 Ferrostatin-1, liproxstatin-1 and antioxidants

Ferrostatin-1 is a first-generation inhibitor of ferroptosis (13). Since the discovery of the anti-ferroptotic effects of this small-molecule compound, ferrostatin-1 has undergone extensive testing in various diseases, including kidney disease. Ferrostatin-1 attenuates cell death in cellular models of kidney dysfunction by inhibiting lipid peroxidation (224). Ferrostatin-1, the first known inhibitor of ferroptosis, also protected Gpx4-/- cells from I/R-induced renal damage (27). Furthermore, a study demonstrated that ferrostatin-1 could effectively prevent ferroptosis cell death and inhibit the proliferation of Pkd1-deficient cells in the kidneys of Pkd1 mutant ADPKD mice. Moreover, it was observed that the increased levels of lipid peroxidation product in Pkd1-deficient mice enhanced the proliferation of the surviving Pkd1 mutant cells by activating Akt, S6, Stat3, and Rb signaling pathways during ferroptosis (204). Pretreatment of ferrostatin-1 has significantly alleviated cisplatin-induced renal injury via trans-regulation of vitamin D receptor (VDR)/GPX4 (204). Furthermore, studies have demonstrated that ferrostatin-1 can reduce renal fibrosis in DN and lessen the injury to renal tubular basement membranes in diabetic db/db mice models by modulating the HIF-1α/HO-1 pathway (242). Another study showed that ferrostatin-1 could also prevent ferroptosis by inhibiting the ZIP14 initiation of ferroptosis in DN, suggesting a promising novel therapeutic strategy for the treatment of DN (225). Furthermore, treatment with ferrostatin-1 has been shown to protect renal function and reduce histologic injury, oxidative stress, and tubular cell ferroptosis in a mouse model of FA-induced AKI. Ferrostatin-1 also inhibited the upregulation of interleukin-33 (IL-33) and the infiltration of macrophages (182). A subsequent study demonstrated the effectiveness ferrostatin-1 in mitigating oxidative stress-induced ferroptosis in renal tubular epithelial cells through the modulation of the PGE2 pathway (226). Furthermore, hemopexin and hemopexin accumulate in the proximal tubular cells and exacerbate kidney injury in both the cisplatin-induced and the unilateral kidney IRI models of AKI. Ferrostatin-1 inhibits this harmful effect of Hb and hemopexin in proximal tubular cells, suggesting the involvement of iron toxicity in the mechanism underlying hemopexin-mediated injury (243).

Several studies have revealed that the *in vivo* efficacy ferrostatin-1 is comparatively lower than its *in vitro* activity due to its limited stability in plasma (227). UAMC-3203, a novel inhibitor derived from ferrostatin-1, has been developed to function as a stronger and more stable ferroptosis inhibitor. These analogues exhibit superior properties to ferrostatin-1, demonstrating *in vivo* efficacy and representing novel compounds with therapeutic potential in ferroptosis-driven disease models, including kidney disease (227, 228).

Liproxstatin-1 was initially identified as a specific inhibitor of ferroptosis in GPX4 knockout cells (27). Subsequent research showed that liproxstatin-1 can inhibit ferroptosis by inducing GPX4 expression, reducing cyclooxygenase-2 (COX-2) expression and inhibiting the kidney inflammation activation in the IRI model (129). Furthermore, another study demonstrated that liproxstatin-1 ameliorates ferroptosis-mediated renal IRI with decreased lipoperoxidation (LPO), MDA and lactate dehydrogenase (LDH) levels while simultaneously increasing the GSH level (229). Furthermore, liproxstatin-1 significantly prolonged the erythrocyte enucleation process in a Fe(II)-dependent renal tubular injury model. The study also highlights the involvement of ferroptosis in various biological processes, including embryonic erythropoiesis and the aging process. Therefore, liproxstatin-1 is suitable for further investigation and evaluation (230). Furthermore, liproxstatin-1 treatment reduced serum amylase levels, as well as tumor necrosis factor-α (TNF-α), interleukin-6 (IL-6), Cr and BUN levels in rats with SAP. Additionally, it reduced lipid peroxidation in the kidneys and mitigated histopathological damage in pancreatic and renal tissues. These findings unequivocally demonstrate the therapeutic potential of liproxstatin-1 in the treatment of SAP-induced AKI (187).

Another common antioxidant in kidney disease is Tectorigenin, which exhibits antioxidant activity (193). The protective effect of tectorigenin against obstructive nephropathy is elucidated in a study that demonstrates its inhibitory effect on Smad3-mediated ferroptosis and fibrosis in a UUO mouse model (231).

Considering the potential contribution of ferroptosis and necroptosis to kidney disease mortality, employing a combination strategy could enhance the efficacy of kidney disease management (9). Nec-1f can target receptor-interacting protein kinase 1 (RIPK1) and ferroptosis in cell lines, improving survival in a mouse model of renal IRI (176).

### 5.2 Iron chelators and iron chelation therapy

Iron chelators bind to both circulating and intracellular iron, facilitating the elimination of iron through urinary and fecal routes. Most animal models have shown the efficacy of iron chelation therapy in mitigating kidney diseases.

Deferoxamine (DFO) was the initial iron chelator approved by the Food and Drug Administration (FDA). DFO binds to iron in the circulation, forming the DFO-iron complex (known as ferrioxamine), which is eliminated through urine and feces (183). The upregulation of COX-2, 4-HNE, TNF-α, MCP-1, and IL-6 levels induced by cisplatin was hindered by DFO. DFO also alleviated cisplatin-induced kidney injury and renal dysfunction. The protective effect of DFO against cisplatin-induced nephrotoxicity (CIN) may be partially attributed to the inhibition of ferroptosis (232). Moreover, in mice with UUO, DFO effectively averts renal tubulointerstitial fibrosis by modulating TGF-β/Smad signaling, mitigating oxidative stress, and suppressing inflammatory responses (233). Another study demonstrated that treatment with DFO effectively suppressed renal injury and fibrosis by influencing ferroptosis induction in the remnant kidney following partial nephrectomy, thereby elucidating the underlying pathogenesis (234).

In a CKD rat model, DFX administration was found to alleviate renal fibrosis by suppressing the TGF-1/Smad3 signaling pathway, reducing inflammation, and mitigating oxidative stress (201). A subsequent study found that DFX and vitamin D3 co-therapy mitigates iron-induced renal injury in rats by effectively modulating cellular anti-inflammatory, anti-oxidative stress, and iron regulatory pathways (236).

Deferiprone (DFP), a novel iron chelator administered orally, has been shown to attenuate AKI by inhibiting ferroptosis in two studies: CIN in rats (235) and an aluminum chloride-induced AKI model in mice (237). A recent study found that combination therapy with DFP and DFO significantly enhanced renal protection compared to DFP alone (237).

Cysteine is the rate-limiting precursor in GSH biosynthesis. Studies have demonstrated that adding cystine or cysteine to cell culture media *in vitro* effectively inhibits ferroptosis (115). N-acetylcysteine (NAC) can improve the bioavailability of cysteine and contribute to positive outcomes in kidney disease (238). NAC could ameliorate CIN in mice by inhibiting the activation of kidney inflammation and the complement system, thereby exerting protective effects (239). Moreover, NAC treatment has effectively decreased circulating markers of oxidative stress and lowered serum creatinine levels. In addition, patients treated with NAC had a significantly lower incidence of severe acute kidney injury than those receiving a placebo (238, 240, 244). These findings strongly suggest that NAC holds promising clinical potential for kidney disease.

### 5.3 Other compounds that can affect kidney ferroptosis

Moreover, there are additional compounds that can effectively suppress kidney ferroptosis by targeting alternative molecular pathways. For example, our study shows that the novel third-generation ferrostatin (SRS 16-86) is more stable and potent than the first-generation compound ferrostatin-1. Ferrostatin-1 protects against IRI by inhibiting mitochondrial permeability transition and preventing postischemic and toxic renal necrosis in ferroptosis (120). Moreover, ACSL4 inhibitor rosiglitazone significantly improved renal function inhibited kidney damage, and decreased inflammatory factors and immune cell infiltration in the kidney of the I/R-induced AKI mice (128). In streptozotocin (STZ) induced DN mice, the inhibition of NF-kB activation and MCP-1 expression by rosiglitazone has been shown to alleviate DN by preventing ferroptosis (241). However, rosiglitazone has been restricted due to adverse reactions in clinical trials (245). Furthermore, treatment with ciclopirox olamine (CPX-O), an iron chelator, suppressed ferritin accumulation in ADPKD and induced ferritinophagy in an iron-independent manner. Consequently, this led to a decrease in cyst growth in ADPKD mice (246). In a mouse model of RCC, it has been observed that L-buthionine-S and R-sulfoximine (BSO) can inhibit the GSH/GPX pathway, leading to the prevention of lipid peroxidation and ferroptotic cell death (210). Furthermore, artesunate effectively inhibits the growth of sunitinib-resistant RCC by inducing cell cycle arrest and ferroptosis (247). Furthermore, administering additional iron-binding compounds such as apotransferrin and neutrophil gelatinase-associated lipocalin has been demonstrated to mitigate the severity of renal injury following IRI (248, 249).

Although numerous drugs targeting ferroptosis have demonstrated promising outcomes in preclinical studies involving cells and animals, clinical trials for kidney disease remain scarce. Notably, DFO (NCT00870883, NCT04633889), DFP (NCT01146925, NCT01770652), and NAC (NCT01218178, NCT00780962) have entered clinical trials (244, 250, 251). Future strategies aimed at inhibiting ferroptosis must prioritize high specificity and selectivity to mitigate off-target effects and potential toxicity. Additionally, antioxidant-based approaches to inhibit ferroptosis may inadvertently impact other cell death pathways, such as apoptosis and necroptosis (176, 252).

## 6 Conclusion and future directions

Ferroptosis is a regulated cell death mediated by iron-dependent accumulation of lipid peroxidation. An increasing body of evidence substantiates ferroptosis’s involvement in diverse kidney diseases. However, there are still some questions that need to be addressed.

Firstly, the crucial mechanisms that regulate kidney ferroptosis still need to be determined. Furthermore, the absence of specific biomarkers for ferroptosis has persisted for an extended period. Therefore, it is imperative to identify distinct markers that can accurately predict the occurrence of ferroptosis in kidney diseases. Thirdly, it has been discovered that various genes are involved in regulating ferroptosis. Therefore, there is an urgent need to develop effective strategies specifically targeting ferroptosis to prevent and treat kidney diseases associated with this process. Furthermore, despite the demonstrated improvement in renal function in animal models through selective inhibition of ferroptosis, there have been no clinical trials to date using ferroptosis-specific inhibitors for the treatment of kidney disease. Therefore, it is necessary to further investigate the effectiveness of these inhibitors through clinical trials. Finally, it is imperative to investigate the molecular mechanisms underlying the interplay between ferroptosis and other types of cell death in the progression of kidney diseases. This exploration holds promise for developing novel treatment strategies targeting diverse forms of cell death in kidney diseases.

In summary, ferroptosis is essential in the pathogenesis of kidney diseases. Excess-free reactive iron can result in tissue damage, thus leading to the widespread recommendation of iron chelation therapy for the treatment of patients with kidney disease associated with iron overload. Further study will propose an effective targeted-ferroptosis strategy for the treatment of kidney diseases.
